# Metabolic consequences of interleukin-6 challenge in developing neurons and astroglia

**DOI:** 10.1186/s12974-014-0183-6

**Published:** 2014-11-06

**Authors:** Jacquelyn A Brown, Stacy D Sherrod, Cody R Goodwin, Bryson Brewer, Lijie Yang, Krassimira A Garbett, Deyu Li, John A McLean, John P Wikswo, Károly Mirnics

**Affiliations:** Department of Psychiatry, 465 21st Avenue South, Vanderbilt University, Nashville, TN 37232 USA; Vanderbilt Institute for Integrative Biosystems Research and Education, 6809 Stevenson Center, Vanderbilt University, Nashville, TN 37235 USA; Department of Physics and Astronomy, 6301 Stevenson Center, Vanderbilt University, Nashville, TN 37235 USA; Department of Chemistry, 5421 Stevenson Center, Vanderbilt University, Nashville, TN 37235 USA; Department of Mechanical Engineering, 333 Olin Hall, Vanderbilt University, Nashville, TN 37235 USA; Department of Biomedical Engineering, 5824 Stevenson Center, Vanderbilt University, Nashville, 37235 TN USA; Department of Molecular Physiology and Biophysics, 702 Light Hall, Vanderbilt University, Nashville, TN 37232 USA; Vanderbilt Kennedy Center for Research on Human Development, 110 Magnolia Circle, Vanderbilt University, Nashville, TN 37203 USA; Department of Psychiatry, University of Szeged, 6725 Szeged, Hungary

**Keywords:** glycerophospholipid, immune activation, interleukin, kynurenine, metabolomics, microfluidics, ultra-performance liquid chromatography - ion mobility - mass spectrometry

## Abstract

**Background:**

Maternal immune activation and subsequent interleukin-6 (IL-6) induction disrupt normal brain development and predispose the offspring to developing autism and schizophrenia. While several proteins have been identified as having some link to these developmental disorders, their prevalence is still small and their causative role, if any, is not well understood. However, understanding the metabolic consequences of environmental predisposing factors could shed light on disorders such as autism and schizophrenia.

**Methods:**

To gain a better understanding of the metabolic consequences of IL-6 exposure on developing central nervous system (CNS) cells, we separately exposed developing neuron and astroglia cultures to IL-6 for 2 hours while collecting effluent from our gravity-fed microfluidic chambers. By coupling microfluidic technologies to ultra-performance liquid chromatography-ion mobility-mass spectrometry (UPLC-IM-MS), we were able to characterize the metabolic response of these CNS cells to a narrow window of IL-6 exposure.

**Results:**

Our results revealed that 1) the use of this technology, due to its superb media volume:cell volume ratio, is ideally suited for analysis of cell-type-specific exometabolome signatures; 2) developing neurons have low secretory activity at baseline, while astroglia show strong metabolic activity; 3) both neurons and astroglia respond to IL-6 exposure in a cell type-specific fashion; 4) the astroglial response to IL-6 stimulation is predominantly characterized by increased levels of metabolites, while neurons mostly depress their metabolic activity; and 5) disturbances in glycerophospholipid metabolism and tryptophan/kynurenine metabolite secretion are two putative mechanisms by which IL-6 affects the developing nervous system.

**Conclusions:**

Our findings are potentially critical for understanding the mechanism by which IL-6 disrupts brain function, and they provide information about the molecular cascade that links maternal immune activation to developmental brain disorders.

**Electronic supplementary material:**

The online version of this article (doi:10.1186/s12974-014-0183-6) contains supplementary material, which is available to authorized users.

## Background

Maternal immune activation (MIA) strongly contributes to the pathophysiology of multiple brain disorders in the offspring, including schizophrenia [1], autism [2,3], major depression [4] and multiple sclerosis [5]. Importantly, mounting evidence suggests that altered immune status is not merely a reaction to a primary disease process, but an etiological contributor to the disease itself [6,7]. It has been well documented that disturbed cytokine levels can cause long-term behavioral changes. For example, in animal models, maternal infection with influenza virus during pregnancy leads to long-lasting microanatomical, neurochemical, and behavioral changes in the brains of the offspring [8,9]. However, such changes are not mediated directly by the viral infection [10]. Rather, MIA appears to be the key [11], as offspring of mothers exposed to polyinosinic:polycytidylic acid (polyI:C, a synthetic cytokine-inducing immunostimulant) show deficits similar to those of offspring whose mothers were exposed to virus infection [12].

MIA triggers a complex immune response that ultimately alters the developmental trajectory of the fetal brain [9]. Cytokines play a critical role in MIA, and in particular, interleukin-6 (IL-6) induction appears to be responsible for many of the long-lasting behavioral changes seen in MIA-born offspring [13,14]. IL-6, a pro-inflammatory cytokine with a profound effect on brain function [15,16], is elevated as a result of various MIA paradigms, and exposure to IL-6 gives rise to a similar behavioral phenotype that is observed in offspring of mothers exposed to flu, lipopolysaccharide (LPS), or polyI:C [12–14]. Furthermore, blocking IL-6 effects with anti-IL-6 antibody during influenza-virus infection, polyI:C exposure, or IL-6 challenge prevents the deleterious effects of MIA [13], thus suggesting that the IL-6 inflammatory cascade is a critical (and at least partially causal) mediator of the deleterious effects observed in the offspring.

Within the central nervous system, cells communicate through multiple mechanisms, including synaptic contacts, active secretion of proteins, and release of metabolites. The non-synaptic communication between the various brain cell types is extremely important during development, and the various chemical gradients are critical for brain patterning, neuronal differentiation and migration, laminar development and establishment of the synaptic framework [17]. Thus, the ability to study the molecular secretions in the developing brain in a high-throughput, unbiased way provides insight into typical fetal brain development and pathological processes that result in developmental disorders. Custom-made, microfabricated bioreactors (such as microfluidic chambers) offer the opportunity to maintain tissue explants and small numbers of primary cells in a close-to-physiological environment, wherein the extracellular volume and fluid interconnectivity between cellular compartments are sufficiently optimized for studying paracrine and autocrine signaling phenomena without dilution [18,19]. This, coupled with ultra-performance liquid chromatography - ion mobility - mass spectrometry (UPLC-IM-MS), provides an opportunity to study the exometabolomic features of numerous cell types under tightly controlled conditions [20] and with narrow temporal resolution.

Considering these strengths, we have applied microfluidic-based culturing of embryonic neurons and glial cells to study their exometabolomic activity under resting conditions and upon stimulation with interleukin-6. In a comprehensive comparison of baseline and IL-6-induced changes across these two cell types, we putatively identified metabolites and molecular pathways that might play a critical role in the pathophysiology of neurodevelopmental disorders.

## Methods

### Experimental animals

C57 black male and female mice were kept in mating for 3 days. E0 was defined as time of vaginal plug. At E15 pregnant females were euthanized with isofuran overdose and embryos removed. The ventral midbrain was isolated as previously described [21]. All procedures were in accordance with IACUC standards and were approved by Vanderbilt’s Institutional Animal Care and Use Committee for use in this study.

### Neuronal and astroglial cell cultures

Neuronal cultures were prepared as previously described using Hibernate-E dissection media [22]. The ventral midbrain of each E15 embryo was dissociated in Hank’s balanced salt solution (HBSS) containing 1% papain and 5 U mL^−1^ DNase (Worthington Biochemicals, Lakewood, NJ, USA) and transferred to a solution containing 1% ovomucoid protease inhibitor (Worthington Biochemicals, Lakewood, NJ, USA). After dissociation, neurons and astroglia were separated by magnetic cell sorting using established protocols [23]. Dissociated cells were immuno-enriched using a PNCAM antibody (Miltenyi Biotec, Bergisch Gladbach, Germany), thus positively selecting for neurons and allowing astroglia to be collected in the flow-through. This purification method achieved approximately 94% purity in cell culture. Following centrifugation, purified neurons were resuspended in neural basal (NB) culture media (supplemented with B-27 and 2 mM L-glutamine) and loaded into poly-D-lysine (10 μg ml^−1^) and laminin (9.6 μg ml^−1^)-coated microfluidic chambers. Primary astrocyte cultures were reconstituted and maintained in minimum essential medium (MEM) (supplemented with 10% albumin and N-2) for 3 days. On day 4, cell culture media was exchanged for NB culture media (supplemented with B-27 and 2 mM L-glutamine) for the following 3 days [24–26]. Microfluidic chambers were loaded with approximately 200,000 cells/well and cell cultures were maintained at a constant gravity-fed flow for 6 days prior to exposure.

### Exposure to IL-6

On the 6^th^ day, the above-described culturing media was replaced with media containing 100 ng ml^−1^ IL-6 (Shenandoah Biotech, Warwick, PA, USA) for 2 hours. Following media replacement, effluent was collected for 2 hours, collecting a total flow-through volume of 60 μl/sample. Three biological replicates for each condition were collected. (*Experimental conditions*: Neuronal cultures, Neuronal cultures + IL-6, Astroglial cultures, Astroglial cultures + IL-6; *Controls:* Empty microfluidic chamber containing no cells, Empty microfluidic chamber no cells + IL-6). Each UPLC-IM-MS measurement was performed in triplicate (technical replicates).

### Microfluidic chambers

Microfluidic devices were fabricated using standard soft lithography methods [27,28] as previously described [29–31]. First, a master mold was formed using a negative SU-8 photoresist. Spin-coating SU-8 2100 (Microchem, Newton, MA, USA) on a silicon wafer at 1500 RPM resulted in a uniform layer of photoresist approximately 200-μm thick. Standard photolithographic methods were used to pattern the desired microchannel features into the SU-8. Briefly, the SU-8 film was exposed to UV light through a 20,000 DPI printed transparency mask (CAD-Art, Bandon, OR, USA), baked for 2 hours at 95°C, and processed with SU-8 developer to yield a 3D relief of the 2D pattern on the mask. After fabrication of the mold, liquid polydimethylsiloxane (PDMS) pre-polymer (Dow Corning, Midland, MI, USA) was mixed with its curing agent (10:1 ratio) and poured over the mold. The PDMS was then degassed for approximately 1 hour and cured in a 70°C oven for at least 2 hours. Following curing, the PDMS layer was removed from the SU-8 mold, and 5-mm diameter holes were punched in the inlet and outlet of each microfluidic channel. Air plasma bonding was then used to attach the PDMS layer to a glass cover slip (VWR Vista Vision, Suwanee, GA, USA). After bonding, Pyrex cloning cylinders (Fisher Scientific, Pittsburgh, PA, USA) were adhered to the inlet/outlet regions of each channel to form small reservoirs to load and remove cells and culture media. Prior to use, individual microfluidic channels were stored in deionized water.

**Figure 1:**
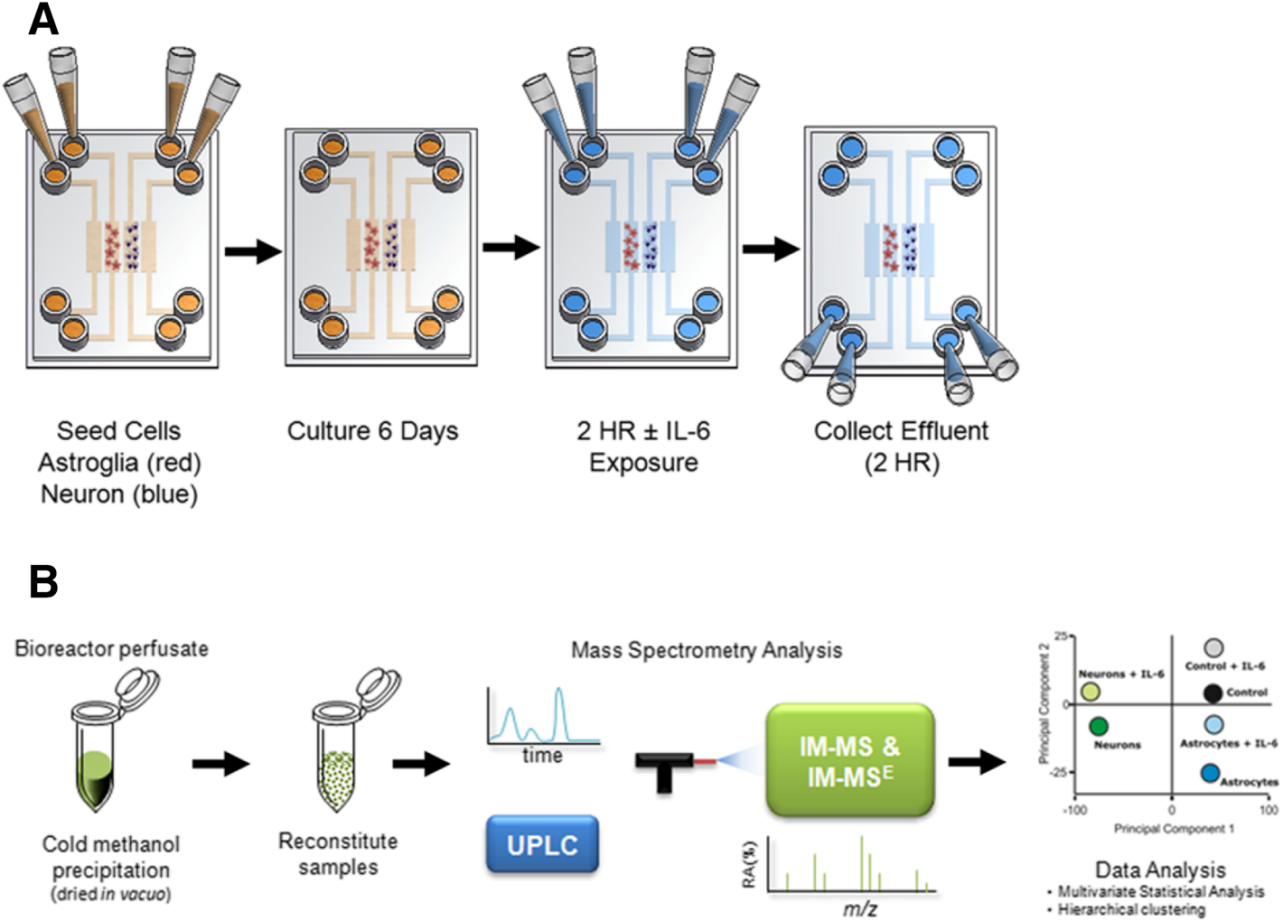
**Experimental design and analysis strategy. (A)** Microfluidic devices or bioreactors were seeded with neurons or astroglia for 6 days prior to IL-6 exposure. **(B)** Illustration of the sample preparation method for bioreactor perfusate samples (control ± IL-6, astroglia ± IL-6, and neurons ± IL-6). Samples were prepared for exometabolomic analyses and analysis performed using an ultra-performance liquid chromatography-ion mobility-mass spectrometer.

Microfluidic devices consisted of four separate microchannels, each having an inlet and outlet channel and one cell culture chamber region (Figure 1A). The devices were designed to reduce flow velocity by expanding the cell culture chamber. The larger cell culture chamber, with dimensions of 5,400 μm (*length*) × 800 μm (*width*) × 200 μm (*height*), facilitates cell attachment and has a total volume of approximately 0.86 μL. A PDMS wall separates the four chambers, ensuring that no cross talk occurs among cell culture regions.

### Sample preparation for mass spectrometry

Metabolites were prepared for ion mobility - mass spectrometry (IM-MS) analysis by concentrating *in vacuo* (SpeedVac concentrator, Thermo-Fisher) and reconstituting in 60 μL of 90% acetonitrile, 10% H_2_O, and 20 mM ammonium acetate (pH = 9). Quality control samples were prepared by combining equal volumes (15 μL) of each sample type.

### Mass spectrometry and data analyses

UPLC-IM-MS and data-independent acquisition (MS^E^) were performed on a Waters Synapt G2 HDMS (Milford, MA, USA) mass spectrometer equipped with a Waters nanoAcquity UPLC system and autosampler (Milford, MA, USA). Metabolites were separated on a 1 mm × 100 mm hydrophilic interaction column packed with 1.7-μm, 13-nm ethylene bridged hybrid (BEH) particles (Waters, Milford, MA, USA). Liquid chromatography was performed using a 20-minute gradient at a flow rate of 90 μL min^−1^ using solvent A (10% H_2_O (v/v) with 10 mM ammonium acetate at pH 9 in acetonitrile) and solvent B (100% H_2_O with 10 mM ammonium acetate at pH 9). A 3-min wash period (99% solvent A) was performed prior to any gradient changes. After 3 min, solvent B increased to 75% over 12.5 min and up to 50% in 15 min. The column was re-equilibrated to 99% solvent A for 5 min after each run. Typical IM-MS analyses were run using resolution mode, with a capillary voltage of 3.5 kV, source temperature at 120°C, sample cone at 5, source gas flow of 400 mL min^−1^, desolvation temperature at 400°C, He cell flow of 180 mL min^−1^, and an IM gas flow of 90 mL min^−1^. The data were acquired in positive ion mode from 50 to 1700 Da with a 0.3 s scan time; full-scan data were mass corrected during acquisition using an external reference consisting of 3 ng mL^−1^ solution of leucine enkephalin infused at a flow rate of 7 μL min^−1^. All analytes were analyzed using MS^E^ with an energy ramp from 10 to 45 eV.

### Data analysis

The integrated intensity of each chromatographically resolved m/z peak was log_2_ transformed. Differences in these peak areas were determined using the average logarithmic ratio (ALR = mean_EXP_ - mean_CNTR_). Statistical significance was determined using unpaired two-tailed Student’s t-test. Both biological and technical replicates were used in these calculations. An m/z species was considered differentially expressed when it met the dual criteria of absolute value |ALR| >0.585 (50% change) and *P* value <0.05. Direction of change was defined as ALRs >0.585 = produced ALRs < -0.585 = consumed. The log_2_ peak area values of species with differential levels between the experimental and control samples were subjected to a two-way unsupervised hierarchical clustering analysis based on Euclidian distance using GenePattern software [32].

### Identification of analytes by ion mobility - mass spectrometry

The raw data acquired were converted to mzXML files using ProteoWizard msconvert [33]. Following conversion, mzXML files were analyzed using XCMS in the statistical package R Studio (v 0.97.449) to pick and align features (that is, retention time (RT) - mass-to-charge (*m/z*) ratio pairs). XCMS was used with default settings except for rector (method = ‘obiwarp’). Data were then normalized to the summed total ion intensity per chromatogram, with the total ion count normalized to 10,000 counts. The resulting processed data matrix was introduced into uMetrix (Version 2.0.0) EZ Info multivariate statistical analysis software for principal component analysis (PCA) with Pareto scaling.

Metabolite peak identifications were putatively assigned using both accurate mass measurements and MS/MS analysis. Ion mobility separations were utilized to isolate precursor ions, correlate product ions, and discern in-source fragmentation. Candidate structures were obtained and interpreted with available biochemical databases, including METLIN [34], the Human Metabolome Database [35], and LIPID MAPS [36] [see Additional files 1, 2, 3, 4 and 5].

### Determination of metabolomics differences

Principal component analysis was performed on expression levels of all measurable analytes using uMetrix (v 2.0.0) EZ Info extended statistics software. Unsupervised, two-way hierarchical clustering (samples versus analytes) was performed on normalized log_2_ analyte levels in GenePattern [32] using Pearson distance. To determine differentially expressed analytes, all analyses were performed on log2-transformed values. Analytes were considered differentially expressed if they met a dual set of criteria of magnitude change and significance (*P* <0.05; │ALR│ >0.585). Additionally, for an analyte to be changed under IL-6 stimulation, it also had to be significantly different (*P* <0.05) from the IL-6 media perfused through the microfluidic chamber containing no cells. These calculations were performed in MS-Excel 2010.

## Results

### Resting astroglia and neurons exhibit distinct metabolic signatures

To investigate the resting-state metabolic signatures of astroglia and neurons, these cells were isolated from E15 mouse embryos using magnetic beads and cultured for 6 days in microfluidic chambers (Figure 1A). On day 6, we collected conditioned media from neuronal and astroglial cells, and as a control, media was perfused through the microfluidic device that contained no cells. Each conditioned media was prepared and analyzed by UPLC-IM-MS (Figure 1B). Following data processing (peak picking and alignment), 2,295 unique features (with each feature having both a mass-to-charge ratio (m/z) and a retention time (RT)) were detected [see Additional file 6: Dataset 1]. To enable study of the metabolic signatures and differences between cell types, the area of each UPLC-IM-MS peak from media for each cell type (neurons and astroglia) was compared to the same features in control media using a paired t-test. In this analysis, 14% (327) of the detectable exometabolites reported significantly different levels (*P* <0.05) between neuronal cultures and control conditions. In contrast, 99% (2,278) of the detectable exometabolites were present at significantly different levels (*P* <0.05) between astroglia and matched controls.

**Figure 2:**
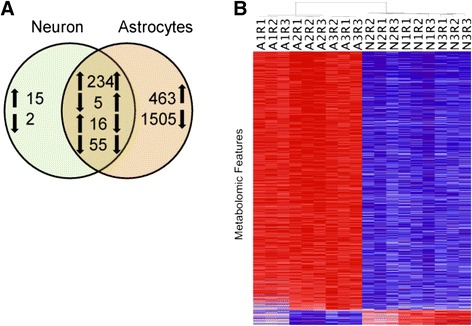
**Unstimulated neurons and astrocytes have a distinct exometabolomic profile. (A)** Venn diagram illustrating the number of features with significant differences between neurons versus control, and astrocytes versus control (*P* <0.05). Numerals denote the number of analytes reporting significantly different levels, while arrows denote directionality of change in comparison to controls (perfused chambers containing no cells). Note that the astrocytes show much higher metabolic activity than neurons, and that 94.8% of the changed neuronal metabolites are also observed in cultured astrocytes. Of the 2,278 species detected in the astrocyte-conditioned media, 69% were consumed, as compared to 19% in the neuronal media. **(B)** Clustering of unstimulated astrocytic and neuronal exometabolomic profiles. Heat map shows two-way unsupervised hierarchical clustering analysis of normalized metabolomics data for the features observed in astrocytes and neurons in their unstimulated state after subtraction of the peak areas for control media (for analytes, [see Additional files 6, 7, 8 and 9]). Columns represent biological and technical replicates (for example, A1R1 - astroglia biological replicate 1 technical replicate 1), rows represent individual chemical species identified by the ultra-performance liquid chromatography - ion mobility - mass spectrometry (UPLC-IM-MS). In individual features, red denotes higher metabolite production in comparison to the other cell type. Note that higher metabolite production was the predominant feature of the astrocytic cultures, and that the clustering separated the biological and technical replicates into two distinct neuronal and astroglial clusters.

Of the 2,278 statistically significant chemical species, 1,576 (69%) were at lower levels in astrocyte-conditioned media than in the control media (three downward arrows in the astrocyte circle in Figure 2A), consistent with the astroglia metabolizing the associated chemical species provided by the original media. For the astroglial media, increased levels of 702 features (31%) relative to the control are indicated by upward arrows and represent production of these species by the astroglia. In contrast, neurons consumed 62 (19%) and produced 265 (81%) species.

However, it is noteworthy that the exometabolic activity observed in neurons showed a large overlap with the species observed in astroglia: of the 327 species consumed or produced by neurons, 310 (94.8%) were common to both the astroglial and neuronal media, with 289 (93.2%) of these showing the same directionality of changes (Figure 2A). We hypothesize that this common neuronal-astroglial exometabolic signature reflects basic homeostatic activity such as glucose utilization, mitochondrial function, and basic protein/lipid turnover.

Next, to directly compare the levels of astroglial species with the neuronal ones, we used a combined paired t-test and magnitude-change criteria applied to the average log_2_ ratio (ALR), such that *P* <0.05 AND│ALR│ >0.585, which selects only species with a >50% difference. Of the original 2,278 species, 872 species met both criteria and revealed that astroglia produced approximately 15× more metabolites than neurons (astroglia = 824, neurons = 48) [see Additional file 7: Dataset 2].

For all technical and biological replicates and each of the 872 species, we computed the deviation of the astroglial and neuronal levels of each sample from the combined mean of that species for all samples. This created a matrix of 872 × 18. We then performed a two-way unsupervised hierarchical clustering on this matrix to produce the self-organized data in Figure 2B. The deviation from the mean was color-coded (red > mean, blue < mean), with the intensity of each color proportional to the magnitude of that deviation. This analysis demonstrates the technical and biological reproducibility of the measurements of these 872 species, and that the unsupervised clustering separates the species by the cell type that conditioned the media. Hence, our data suggest that astroglia and neurons have distinct metabolic signatures under resting conditions, while astroglia have a robust consumption/production metabolic activity and neurons consume/produce fewer metabolites in their resting state.

### Metabolic signatures of neurons and astroglia in response to IL-6 challenge

**Figure 3:**
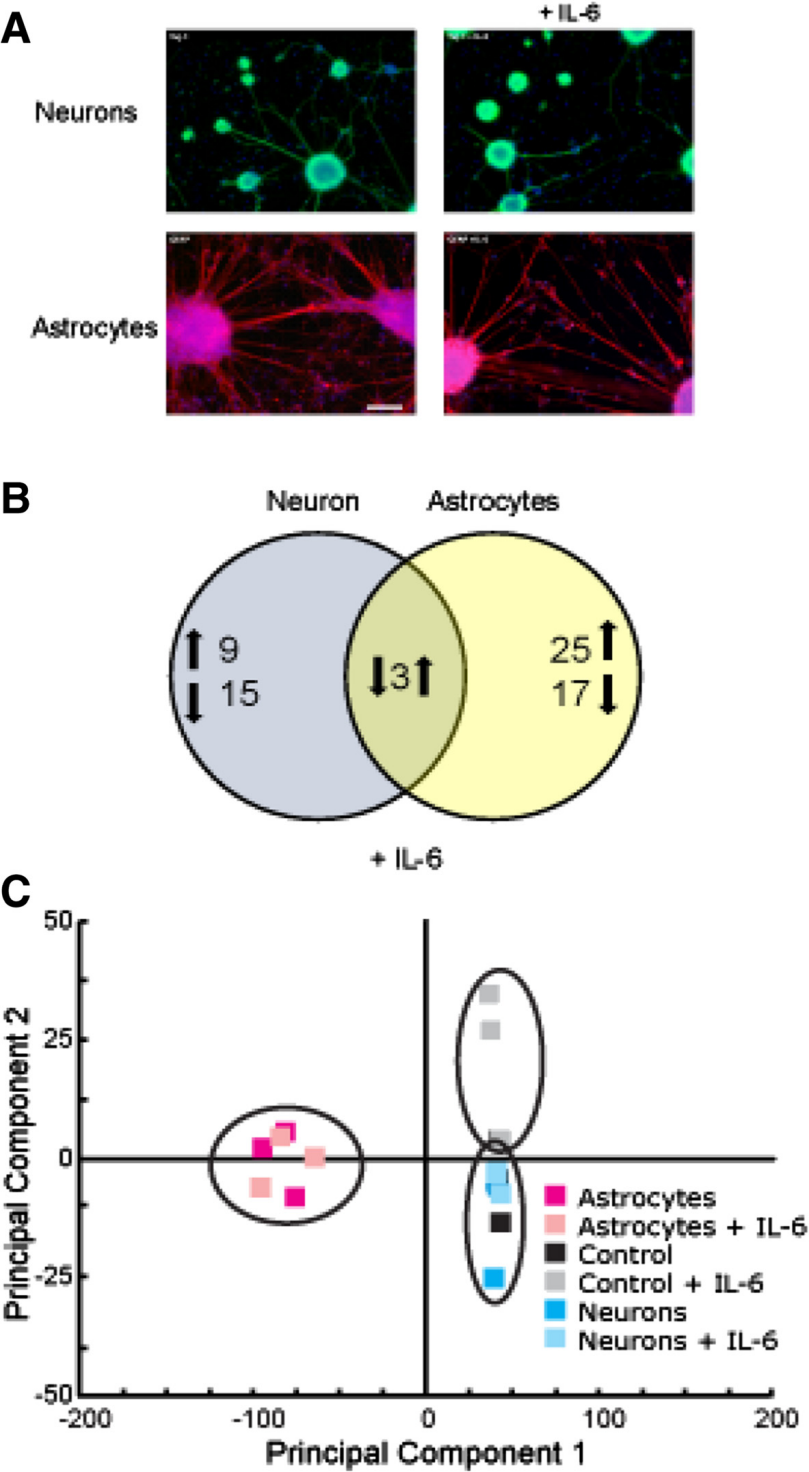
**Effects of IL-6 exposure on astrocytes and neuronal cultures. (A)** Cultured neurons and astrocytes were exposed to IL-6 for 2 hours and allowed to recover for 1 hour prior to imaging. Cells were fixed with 4% PFA and labeled with neuronal marker Tuj-1 or GFAP, an astrocyte marker. Appearance, process length, and cell density were similar to the untreated, matched controls. **(B)** Venn diagram illustrating the number of features with significant differences between IL-6 treatment and matched, sham-treated control cultures (*P* <0.05;│ALR│>0.585, that is*,* >50% of control). Numerals denote the number of features (*m/z* and retention time pairs) with significantly different levels, while arrows denote directionality of change in comparison to controls. Note that the neuronal and astroglial exometabolome response to IL-6 treatment is distinct. **(C)** Global principal component analysis (PCA) of ultra-performance liquid chromatography - ion mobility - mass spectrometry (UPLC-IM-MS) data illustrating that there are distinct metabolic signatures between neuronal and astroglial sample types.

To investigate the metabolic response of each cell type to pro-inflammatory cytokine stimulation, we cultured the isolated astroglia and neurons in microfluidic devices, and cultures were exposed to 100 ng ml^−1^ IL-6 for 2 hours on day 6. Control cells were exposed to vehicle only, and the control device contained no cells but was perfused with media containing IL-6. To address the issue of toxicity for both IL-6 dose and exposure time, neuronal and glial cells ± IL-6 were imaged 1 hour after treatment using β3-tubulin antibody (TUJ-1, a neuronal marker) or glial fibrillary acidic protein (GFAP, astrocyte marker) staining. Assessments did not reveal any change in process length, cell density, or other morphological signs of toxicity (Figure 3A).

The extracellular metabolomic signatures of neuronal and astroglial samples were significantly altered in response to IL-6 stimulation [see Additional files 8 and 9]. In the neuronal cultures, we observed 27 metabolites that changed in response to IL-6 exposure, with nine features exhibiting increased abundances and 15 demonstrating decreased abundances [see Additional files 8 and 9]. The response of the astroglial cultures to IL-6 exposure was more pronounced; of the 45 changed species, 28 species reported increased exometabolomic abundances, while 17 species were observed at reduced levels in the media [see Additional files 8 and 9]. Importantly, the responses of neuronal and astroglial cultures to IL-6 stimulation were quite distinct (Figure 3B): the observed neuronal changes were dominated by exometabolome decreases, while the majority of the astroglial alterations were increases. It is also noteworthy that three secreted species were significantly different in both the neurons and astroglia when compared to non-IL-6 exposed cultures (*m/z* 664.291, RT = 232 s; *m/z* = 760.278, RT = 213 s; *m/z* 748.234, RT = 109 s), with all showing opposite directionality of change between the two cell types [see Additional files 8 and 9]. These data illustrate that the initial, narrow time window for metabolic response of astroglia to IL-6 stimulation was predominantly characterized by an increase in metabolite production, while neurons increased their metabolite consumption.

These data comparisons between neuronal cultures, astroglial cultures, and control conditions demonstrate the powerful capabilities of UPLC-IM-MS analysis without *a priori* knowledge. This is also demonstrated by a principal component analysis (PCA) that distills multi-dimensional data sets (in these data, approximately 2,300 m/z-RT pairs) to principal components that best describe the greatest covariance in the data, thus providing sample grouping or separation based on patterns in the data. Global PCA of processed UPLC-IM-MS data from neurons, astroglia, and controls further confirmed that the global metabolic signatures between neuronal and glial sample types were quite distinct (Figure 3C).

### Identification of selected analytes by ion mobility - mass spectrometry

Peak identifications were performed using accurate mass and ion adduct information for database searching (METLIN [34], LIPID MAPS [36], and the Human Metabolome Database [35]) to produce candidate structures. Candidate structures were then validated using retention time and ion mobility-filtered high-energy data. Fragmentation is performed after the mobility separation, and therefore, product ions retain the mobility of the precursor. This allows untargeted fragmentation of all ions to be filtered into relevant high-energy spectra.

**Figure 4:**
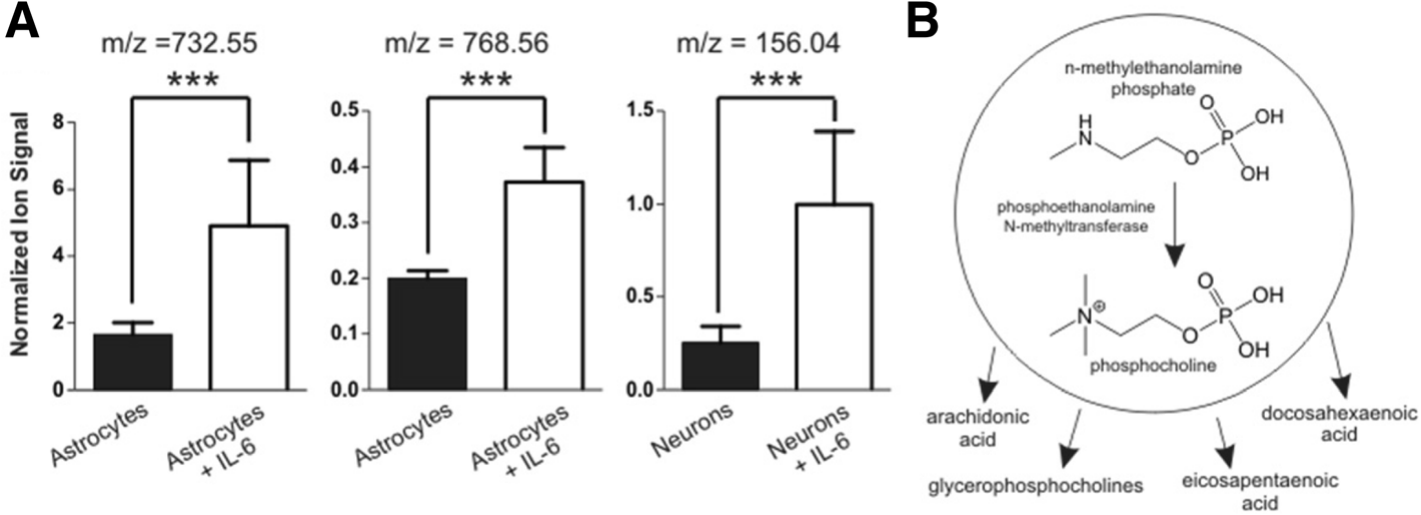
**Activation of glycerophospholipid metabolism in response to IL-6. (A)** Numerous glycerophospholipid metabolites were significantly produced upon IL-6 exposure in both astrocytes and neuronal cultures. Three analytes were putatively identified as diacylglycerophosphocholine (*m/z* 732.55, ALR = 1.57, *P* <0.001), diacylglycerophospho-ethanolamine (*m/z* 768.56, ALR = 1.39, *P* <0.001), and n-methylethanolamine (*m/z* 156.04, ALR = 1.81, *P* <0 .001). Putative identifications were obtained by using mass measurement accuracy, retention time and confirmed based on fragmentation spectra [see Additional files 1, 2, 3, 4 and 5]. **(B)** Illustrates a simplified glycerophospholipid metabolism pathway and shows that n-methylethanolamine is a precursor metabolite to numerous developmentally important downstream metabolites.

From the prioritized list of metabolites altered by IL-6 exposure, we putatively identified three analytes (Figure 4A) that belong to the glycerophospholipid metabolism pathway (Figure 4B). The connection of this pathway to brain IL-6 activation has not previously been described. *N*-methylethanolamine phosphate was observed to be increased in the IL-6-exposed neurons (m/z = 156.065, RT = 314 s, ALR = 1.81, *P* <0.001) in comparison to the sham-treated neuronal cultures. Baseline levels of *N*-methylethanolamine phosphate were significantly higher in the glial cultures compared to those observed in the neuronal cultures. *N*-methylethanolamine phosphate can be produced through the transfer of a methyl group from *S*-adenosyl methionine to phophoethanolamine, catalyzed by phosphoethanolamine methyltransferase. This metabolite has an important biological role in glycerophospholipid metabolism and in development [37]. Importantly, the production of at least two other glycerophospholipid metabolites was detected in the secreted metabolome of IL-6 stimulated astroglia: diacylglycerophosphocholine (m/z = 732.551, RT = 216 s, ALR = 1.57, *P* <0.001) and diacylglycerophospho-ethanolamine (m/z = 768.564, RT = 124 s, ALR = 1.39, *P* <0.001). Glycerophosphocholines are the inactive form of long-chain polyunsaturated fatty acids (LCPUFAs), arachidonic acid (AA), docosahexaenoic acid (DHA), and eicosapentaenoic acid (EPA), and the disturbed metabolism of these species has been previously linked to schizophrenia, major depression, and bipolar disorder [38–42].

**Figure 5:**
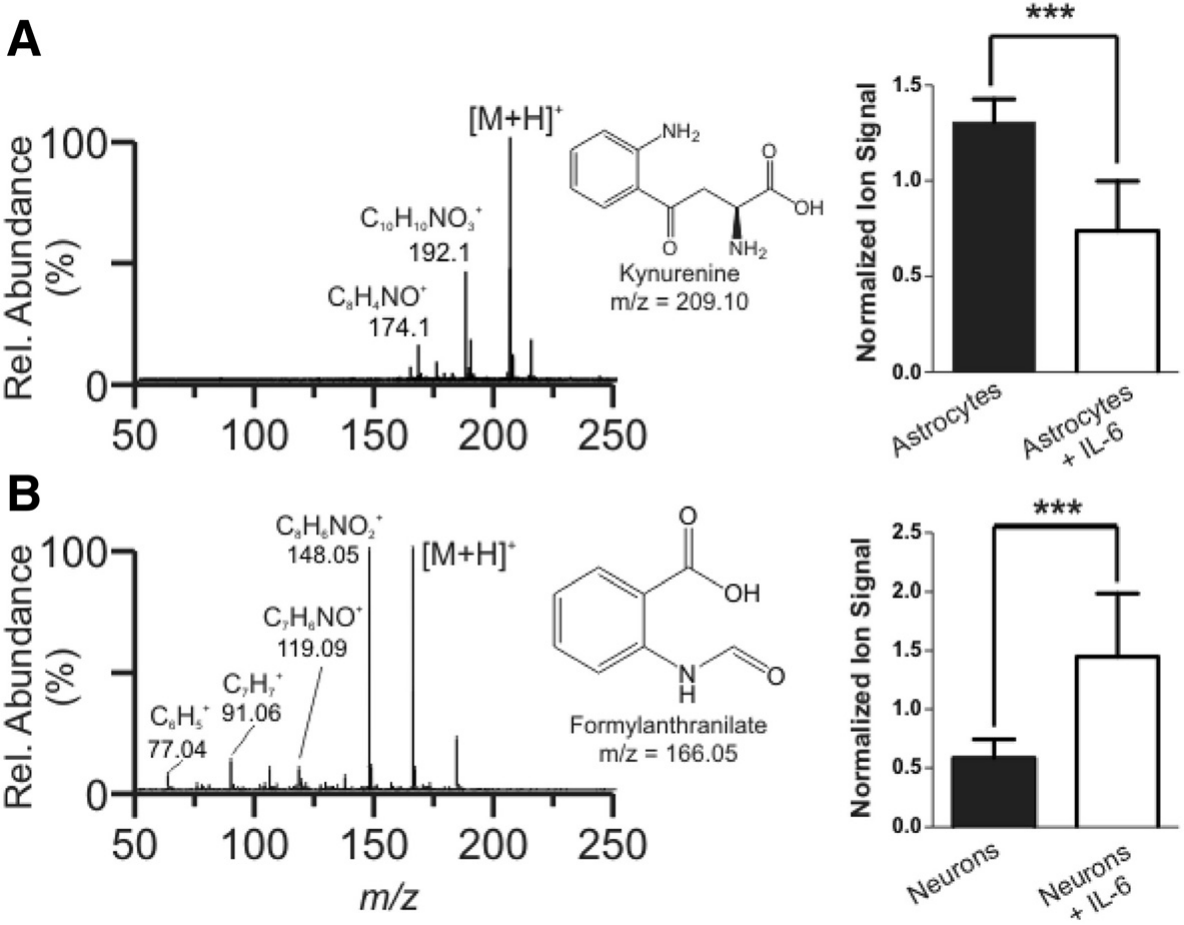
**Alterations to the kynurenine pathway after IL-6 treatment.** High energy (fragmentation) mass spectra for **(A)** *m/z* 209.10 (kynurenine) and **(B)** *m/z* 166.05 (formylanthranilate). Spectra were mobility separated to isolate only product ions for 209 and 166, respectively. **(A)** In astrocyte cultures, we observed a decrease in normalized ion signal after IL-6 treatment, ALR = −0.86, *P* <0.00001, for kynurenine (*m/z* 209.10). **(B)** In contrast, we observed an increase in ion signal for formylanthranilate (m/z 166.05)*,* also a component of the kynurenine pathway, when neuronal cultures were treated with IL-6, ALR = 2.47, *P* <0.001. Taken together, these data show that IL-6 exposure alters both neurons and astrocytes but does so in an opposite manner.

In addition, in both neuronal and astroglial cultures we observed IL-6-induced exometabolome changes in the tryptophan-kynurenine pathway, a pathway strongly modulated by IL-6 activity in the brain. Kynurenine (m/z = 209.10, RT = 78 s) was observed to decrease in astroglial cultures in response to IL-6 exposure (Figure 5A, ALR = −0.86, *P* <0.00001), whereas formylanthranilate (m/z = 166.07, RT =70 s) was observed to increase in neuronal cultures in response to IL-6 exposure (Figure 5B, ALR = 2.47, *P* <0.001). Formylanthranilate is a downstream metabolite in the kynurenine pathway that yields neuroactive intermediates and has implications in the modulation of neurotransmitter systems [43]. Importantly, both formylanthranilate and kynurenine are part of the tryptophan-kynurenine metabolic pathway, and disturbances of this pathway are considered common mediators of genetic and environmental effects in major depressive disorder [44] and appear to contribute to schizophrenia pathophysiology [41,45]. Taken together, these data suggest that glycerophospholipid metabolism and tryptophan-kynurenine metabolite secretions are important (and potentially related) metabolic pathways by which IL-6 inflammation affects the developing nervous system and predisposes it to developing a brain disease.

## Discussion

In traditional culture systems, cells are grown in a volume of media that is large enough to require media changes no more than once per day [46], often diluting the secreted molecules [47,48] to non-detectable levels [19]. Molecular signals can ostensibly be concentrated by prolonged culturing time, but at a cost of significant loss in temporal resolution. This is problematic, since cellular response to cytokine exposure is swift [49] and typical physiological effects of cytokines are characterized by rapid cycles [50]. To address these issues, we implemented a microfluidic culture chamber system with a 30-fold decrease in metabolite dilution when compared to conventional culture methods. This provided a means to increase temporal sampling resolution of the exometabolome, thereby utilizing the resultant increase in the sensitivity to changes in the concentrations of extracellular secretion. This increased sampling rate decreased the probability of analyte degradation and dilution over time. Furthermore, in the future it will allow investigators to perform continuous post-stimulation sampling in narrow time windows, opening the door to reconstruction of temporal cascades of metabolic activity.

Astroglia are well known to communicate with secreted molecules, and astrocyte-conditioned media has proven beneficial for neuronal growth and survival [47]. Thus, it is not surprising that astroglial cultures at baseline produce almost ten times more detectable analytes than neuronal cultures. An unexpected finding, however, was that resting astrocytes depleted the media of 1,576 analytes, or 69% of detected compounds. Identification of these consumed compounds and understanding the process of resource utilization will be important topics for future studies. It is also important to note that while the neurons enriched the conditioned media by only 15 detected analytes, we cannot exclude the possibility that these molecules are critical for neuronal-neuronal, neuronal-glial, or neuronal-microvascular communications [51].

The outcome of the IL-6 stimulation was perhaps the most interesting finding in our dataset. Neuronal and glial response to IL-6 stimulation was quite distinct, non-overlapping, and divergent: astroglia predominantly increased their metabolite production, while neurons primarily consumed resources from the media. We believe that these two metabolic signatures are parts of an essential chemical communication mechanism between neurons and glia under stress, and they perhaps represent an initial neuroprotective response to adverse conditions. In follow-up experiments, we were able to positively identify five distinct metabolites that were decreased in astroglia-conditioned media in response to IL-6 stimulation. These included diacylglycerophosphocholine, diacylglycerophospho-ethanolamine, and l-kynurenine, whereas N-methylethanolamine phosphate and formylanthranilate secretion increased in IL-6-stimulated neuronal cultures.

Diacylglycerophosphocholine and diacylglycerophospho-ethanolamine belong to the class of glycerophospholipids (GPLs), an inactive pool from which bioactive lipids are synthesized [52,53]. Neuronal-astroglial membranes contain several classes of GPLs, which constitute the membrane backbone and define membrane properties such as fluidity and ion permeability [52,54]. GPLs and the complementary bioactive lipid breakdown products have been shown to modulate membrane excitability, monoaminergic neurotransmission, and brain development [39,55]. Recent evidence suggests that GPL metabolism is a critical disturbance across multiple brain diseases, as degradation of GPLs by phospholipase A_2_ can release arachidonic acid and docosahexaenoic acid. The subsequent peroxidation and enzymatic processing of these long-chain polyunsaturated fatty acids produces multiple bioactive lipid mediators, all closely associated with neuronal metabolic pathways involved in the pathophysiology of major depression, schizophrenia, and Alzheimer’s disease [56–58]. Furthermore, plasma phospholipid concentrations have been demonstrated as predictive of memory impairment in elderly adults [59].

In addition to altered GPL metabolism, exposure to IL-6 demonstrates a significant effect on at least two metabolites within the tryptophan-kynurenine pathway. L-kynurenine is the metabolite whose breakdown leads to the generation of bioactive kynurenic and quinolinic acids, which act on the glutamatergic and nicotinic neurotransmission as antagonists and agonists, respectively [60–62]. The tryptophan-kynurenine pathway is intricately linked to inflammatory cytokines through the enzyme of indoleamine 2,3-dioxygenase (IDO), which is the rate limiting step in the degradation of tryptophan into kynurenine and the subsequent formation of downstream metabolites [63,64]. Tryptophan metabolites demonstrate immunomodulatory effects, with increased tryptophan metabolites leading to apoptosis and cell cycle arrest and induction of T regulatory cells [65,66]. Neuroimaging, biomarker, and animal model studies all indicate that disturbances in the tryptophan-kynurenine pathway are a critical pathophysiological event in major depression [38,64,67] and potentially other neuropsychiatric disorders [41,62,68,69]. Our observation of altered kynurenic acid and formylanthranilate levels (both critical components of the tryptophan-kynurenine pathway) in response to IL-6 stimulation suggests that our system is relevant for studying brain disease-related processes and allows future dissection of this molecular pathway under controlled stimulation of astrocytes and neurons. Furthermore, our experimental system has the capability to dissect the secretory interplay between different brain cell types and immune cells, including (but not limited to) astroglia, neurons, microglia, and ependymal, endothelial, and T cells, as well as the study of the blood-brain barrier [70].

Based on our results, we hypothesize that the previously described detrimental effects of IL-6 on developing neurons are at least partially due to the response of astroglia to IL-6, resulting in an altered exometabolomic profile, which in turn has a damaging effect on neurons. We intend to investigate this hypothesis further in follow-up, mechanistic studies which would test the effects the IL-6-induced astroglial exometabolome has on neuronal differentiation, growth, and synapse formation. This investigation, coupled with precise identification and testing the effects of individual metabolites, would lead to a better understanding of neurotoxic and neuroprotective processes that might occur in the developing brain. Future experiments will also encompass defining exometabolomic changes, responses, and interplay between various subclasses of neurons and across the various brain regions, leading to three-dimensional *in vitro* modeling of the brain.

It is also noteworthy that IL-6 response is quite conserved across species [71–73] and that the general CNS development in rodents is governed by the same basic principles as in humans [74]. Thus, we believe that our findings, obtained on developing mouse neurons, are also relevant for human conditions such as schizophrenia and autism. This view is also underscored by human disease findings of elevated cytokines in schizophrenia and autism [6,7,10], as well as the critical role of IL-6 in maternal immune activation models [8–10].

## Conclusions

In summary, our studies utilize microfluidic technology with a superb cell-to-media volume ratio, which is ideally suited for analysis of cell-type-specific metabolic signatures. We find that 1) developing neurons have lower metabolic activity at baseline than astroglia, which show strong production of metabolites; 2) both neurons and astroglia respond to IL-6 exposure in a cell-type-specific fashion; 3) with IL-6 stimulation of each cell type, metabolite production predominantly increases for the astrocytes as compared to sham-treated cells, but decreases for neurons; and 4) disturbances in glycerophospholipid metabolism and tryptophan/kynurenine metabolite secretion are two important mechanisms by which IL-6 might affect the developing nervous system.

## Additional files

Additional file 1:
**Energy spectra for mass 156.04.** Low (top) and high (bottom) energy spectra for mass 156.04, putatively identified as n-methylethanolamine phosphate. The fragmentation spectrum is mobility selected to isolate only product ions for this mass. Additional file 2:
**Energy spectra for mass 209.10.** Low (top) and high (bottom) energy spectra for mass 209.10, putatively identified as kynurenine. The fragmentation spectrum is mobility selected to isolate only product ions for this mass. Additional file 3:
**Energy spectra for mass 166.05.** Low (top) and high (bottom) energy spectra for mass 166.05, putatively identified as formylanthranilate. The fragmentation spectrum is mobility selected to isolate only product ions for this mass. Additional file 4:
**Energy spectra for mass 732.55.** Low (top) and high (bottom) energy spectra for mass 732.55, a fragment of 760.59 and putatively identified as a phosphatidylcholine (PC). The fragmentation spectrum is mobility selected to isolate only product ions for this mass. A characteristic fragment ion of PCs is an ion at m/z 184, which is the phosphatidylcholine head group (structure shown above). The above structure is ONLY an example of the structure of PCs. Additional file 5:
**Energy spectra for mass 768.56.** Low (top) and high (bottom) energy spectra for mass 768.56, putatively identified as a phosphatidylethanolamine (PE). The fragmentation spectrum is mobility selected to isolate only product ions for this mass. A characteristic fragment ion of PEs is the neutral loss of 141, which is the ethanolaminephosphate head group (structure shown above). The above structure is ONLY an example of the structure of PEs. Additional file 6:
**Dataset 1.** Baseline astroglia and neurons, secreted and consumed – all data. Additional file 7:
**Dataset 2.** Neuron versus glial baseline – all data. Additional file 8:
**Dataset 3.** Effect of interleukin-6 – all data. Additional file 9:
**Exometabolomic response of neurons and glia to IL-6 stimulation.** Abbreviations: m/z, mass-to-charge ratio; RT, retention time; NIL6, neurons + IL-6; GIL6, glia + IL-6 stimulation; EIL6, empty chamber + IL-6 containing media; ALR, average Log2 ratio; *P* val, *P* value.

## Abbreviations

AA: arachidonic acid
ALR: average logarithmic ratio
BEH: ethylene bridged hybrid
CNS: central nervous system
DHA: docosahexaenoic acid
EPA: eicosapentaenoic acid
GFAP: glial fibrillary acidic protein
GPL: glycerophospholipid
HBSS: Hank’s balanced salt solution
IDO: indoleamine 2,3-dioxygenase
IL-6: interleukin-6
IM-MS: ion mobility - mass spectrometry
LC: liquid chromatography
LCPUFA: long-chain polyunsaturated fatty acid
LPS: lipopolysaccharide
MEM: minimum essential medium
MIA: maternal immune activation
MS: mass spectrometry
MS^E^: data-independent acquisition mass spectrometry
NB: neural basal
PCA: principal component analysis
PDMS: polydimethylsiloxane
PolyI:C: polyinosinic:polycytidylic acid
RT: retention time
UPLC: ultra-performance liquid chromatography
UPLC-IM-MS: ultra-performance liquid chromatography - ion mobility - mass spectrometry
