# Supplementary figures and images for: Metabolic consequences of interleukin-6 challenge in developing neurons and astroglia
Source: J Neuroinflammation. 2014 Nov 6;11:183. doi: 10.1186/s12974-014-0183-6 (PMC4233071; doi:10.1186/s12974-014-0183-6)

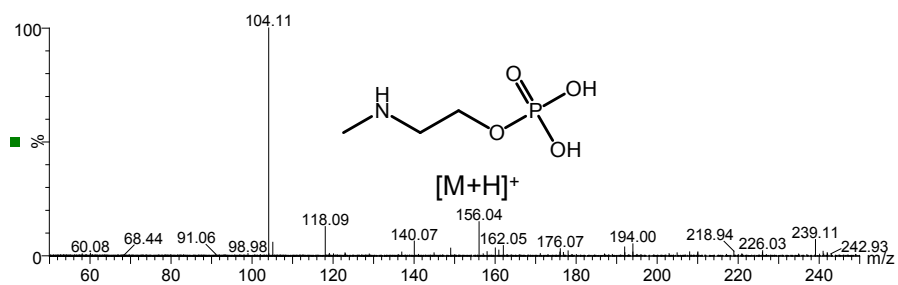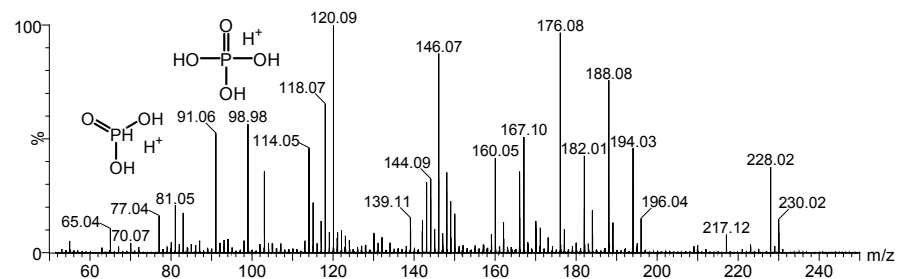

Supplement: Additional file 1: — Energy spectra for mass 156.04. Low (top) and high (bottom) energy spectra for mass 156.04, putatively identified as n-methylethanolamine phosphate. The fragmentation spectrum is mobility selected to isolate only product ions for this mass. [file 12974_2014_183_MOESM1_ESM.pdf]

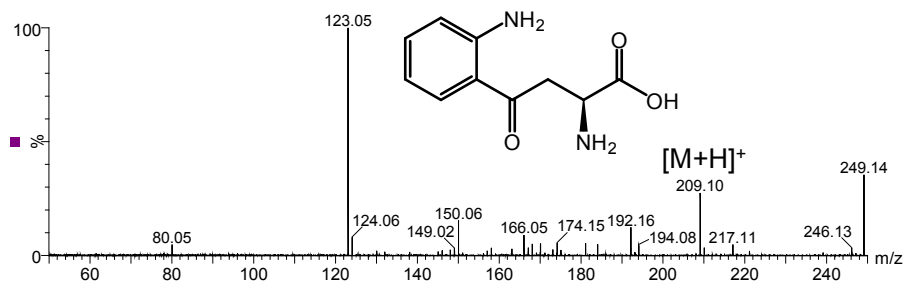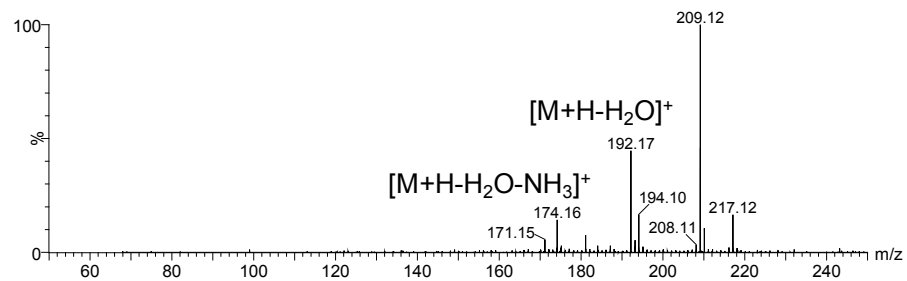

Supplement: Additional file 2: — Energy spectra for mass 209.10. Low (top) and high (bottom) energy spectra for mass 209.10, putatively identified as kynurenine. The fragmentation spectrum is mobility selected to isolate only product ions for this mass. [file 12974_2014_183_MOESM2_ESM.pdf]

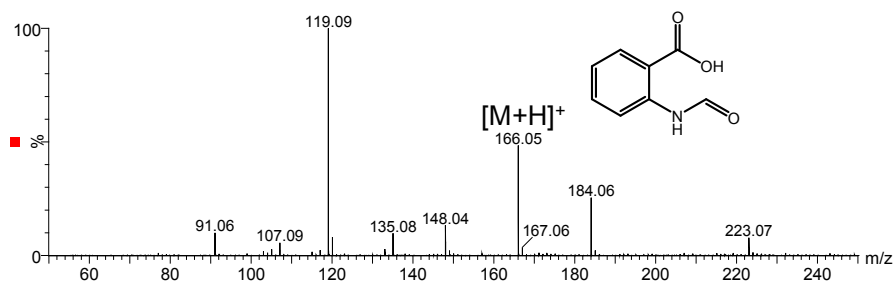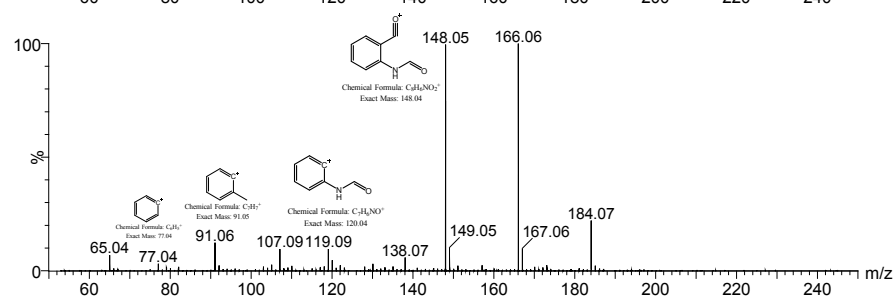

Supplement: Additional file 3: — Energy spectra for mass 166.05. Low (top) and high (bottom) energy spectra for mass 166.05, putatively identified as formylanthranilate. The fragmentation spectrum is mobility selected to isolate only product ions for this mass. [file 12974_2014_183_MOESM3_ESM.pdf]

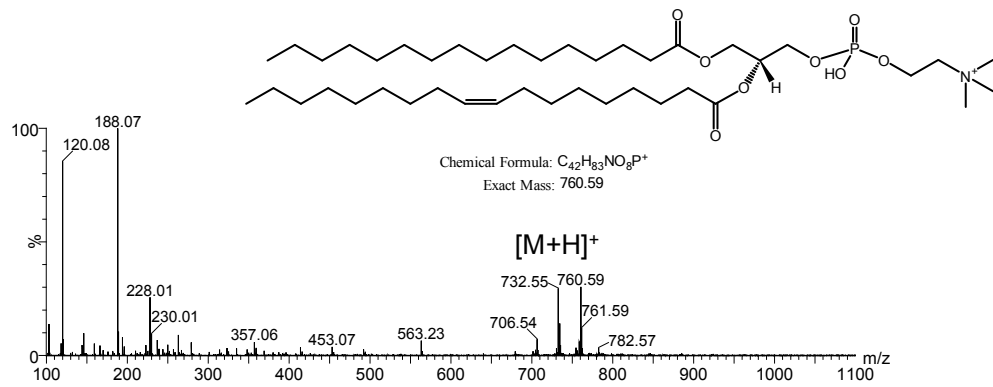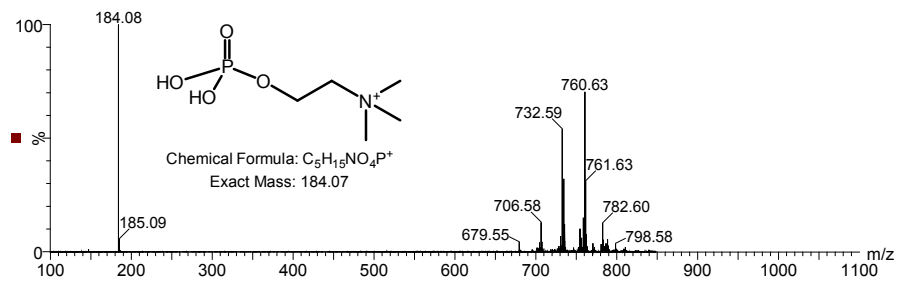

Supplement: Additional file 4: — Energy spectra for mass 732.55. Low (top) and high (bottom) energy spectra for mass 732.55, a fragment of 760.59 and putatively identified as a phosphatidylcholine (PC). The fragmentation spectrum is mobility selected to isolate only product ions for this mass. A characteristic fragment ion of PCs is an ion at m/z 184, which is the phosphatidylcholine head group (structure shown above). The above structure is ONLY an example of the structure of PCs. [file 12974_2014_183_MOESM4_ESM.pdf]

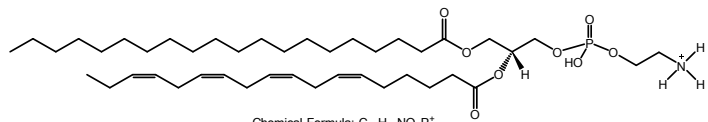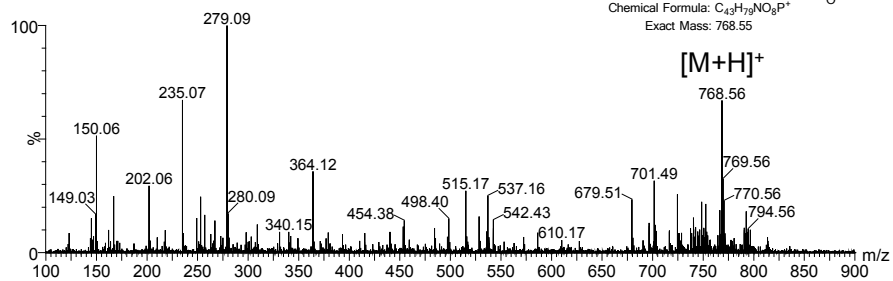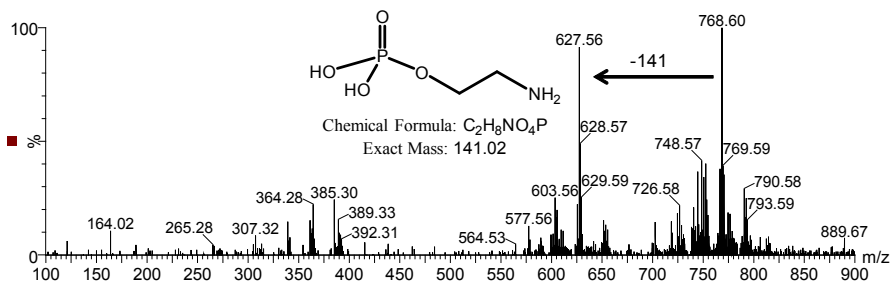

Supplement: Additional file 5: — Energy spectra for mass 768.56. Low (top) and high (bottom) energy spectra for mass 768.56, putatively identified as a phosphatidylethanolamine (PE). The fragmentation spectrum is mobility selected to isolate only product ions for this mass. A characteristic fragment ion of PEs is the neutral loss of 141, which is the ethanolaminephosphate head group (structure shown above). The above structure is ONLY an example of the structure of PEs. [file 12974_2014_183_MOESM5_ESM.pdf]
